# Posterior Cord of Brachial Plexus and Its Branches: Anatomical Variations and Clinical Implication

**DOI:** 10.5402/2013/501813

**Published:** 2013-09-26

**Authors:** Rakhi Rastogi, Virendra Budhiraja, Kshitij Bansal

**Affiliations:** ^1^Department of Anatomy, L.N. Medical College and J.K. Hospital, Sector-C Sarvdharm, Kolar Road, Bhopal, India; ^2^Department of Pediatrics, Subharti Medical College, Meerut, India

## Abstract

*Background*. Knowledge of anatomical variations of posterior cord and its branches is important not only for the administration of anaesthetic blocks but also for surgical approaches to the neck, axilla, and upper arm. The present study aimed to record the prevalence of such variations with embryological explanation and clinical implication. *Material and Method*. 37 formalin-preserved cadavers, that is, 74 upper extremities from the Indian population, constituted the material for the study. Cadavers were dissected during routine anatomy classes for medical undergraduate. Dissection includes surgical incision in the axilla, followed by retraction of various muscles, to observe and record the formation and branching pattern of posterior cord of brachial plexus. *Results*. Posterior cord was formed by union of posterior division of C_5_ and C_6_ roots with posterior division of middle and lower trunk (there was no upper trunk) in 16.2% of upper extremities. Posterior cord of brachial plexus was present lateral to the second part of axillary artery in 18.9% of upper extremities. Axillary nerve was taking origin from posterior division of upper trunk in 10.8% upper extremities and thoracodorsal nerve arising from axillary nerve in 22.9% upper extremities. *Conclusion*. It is important to be aware of such variations while planning a surgery in the region of axilla as these nerves are more liable to be injured during surgical procedures.

## 1. Introduction

Posterior cord of brachial plexus is formed by union of posterior division of upper, middle, and lower trunk of brachial plexus. It lies posterior to, second part of axillary artery. The posterior cord of brachial plexus after giving upper subscapular, thoracodorsal, lower subscapular, and axillary nerve in the axilla continues distally as the radial nerve [1]. Knowledge of the variations of posterior cord and its branches is important for the administration of anaesthetic blocks, surgical approaches to the neck, axilla, and upper arm [2, 3]. The present study describes the variations of posterior cord observed in population from central India.

## 2. Material and Method

The formalin-fixed 37 cadavers, that is, 74 upper extremities constitute the material for study. During routine dissection of axilla and supraclavicular region of medical undergraduates in L.N. Medical College Bhopal, the skin and various muscles were reflected and superficial fascia and deep fascia were separated to visualize the formation and branching pattern of posterior cord.

## 3. Results

**Figure 1:**
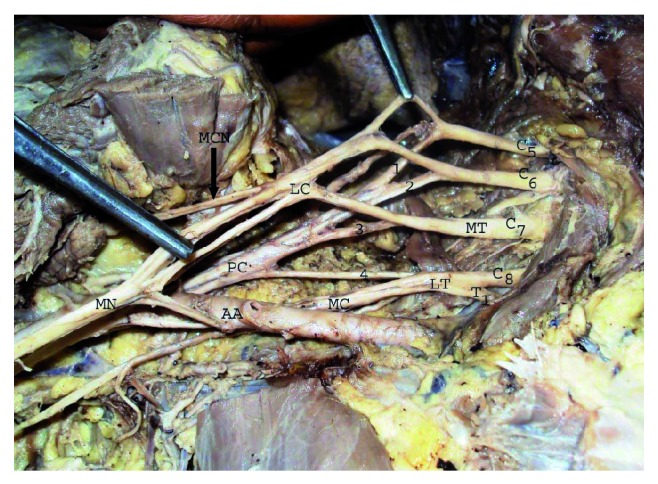
Formation of posterior cord by union of posterior division of C_5_ and C_6_ ventral rami with posterior division of middle and lower trunk. C_5_, C_6_, C_7_, C_8_, T1-ventral rami of spinal nerve, MD-middle trunk, LT-lower trunk, 1,2,3,4-Posterior division of C_5_ and C_6_ ventral rami and middle and lower trunk, LC-lateral cord, PC-posterior cord, MC-medial cord, MCN-musculocutaneous nerve, MN-median nerve, AA-axillary artery.

**Figure 2:**
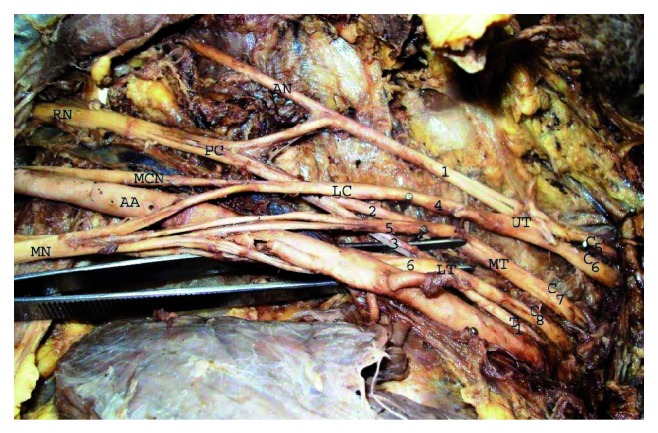
Axillary nerve originates from posterior division of upper trunk and posterior cord in lateral relation of second part of axillary artery. C_5_, C_6_, C_7_, C_8_, T1-ventral rami of spinal nerve, UT-upper trunk, MD-middle trunk, LT-lower trunk, 1,2,3,-Posterior division of upper, middle and lower trunk, 4,5,6-anterior division of upper, middle and lower trunk, LC-lateral cord, PC-posterior cord, MCN-musculocutaneous nerve, MN-median nerve, RN-radial nerve, AN-axillary nerve, AA-axillary artery.

**Figure 3:**
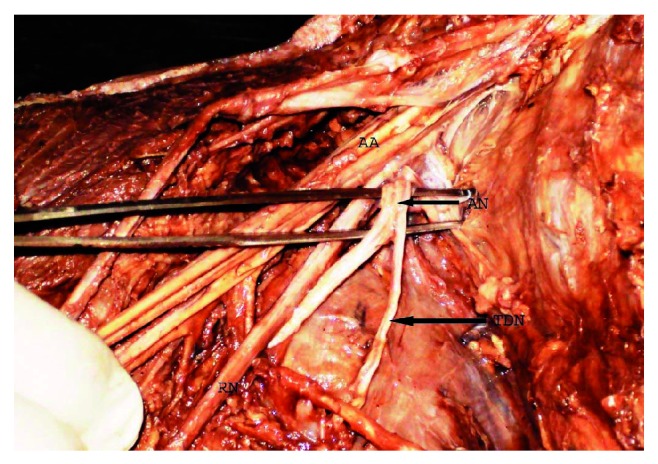
Thoracodorsal nerve originates from axillary nerve. AN-axillary nerve, TDN-thoracodorsal nerve, AA-axillary artery, RN-radial nerve.

We recorded variations in the formation, location, and branching pattern of posterior cord. Posterior cord was formed by union of posterior division of C_5_  and C_6_ roots with posterior division of middle and lower trunk (there was no upper trunk) in 16.2% (12/74) of upper extremities (Figure 1). Posterior cord of brachial plexus was present lateral to the second part of axillary artery in 18.9% (14/74) of upper extremities (Figure 2). Axillary nerve was taking origin from posterior division of upper trunk in 10.8% (8/74) upper extremities (Figure 2) and thoracodorsal nerve arising from axillary nerve in 22.9% (17/74) upper extremities (Figure 3). Upper subscapular, lower subscapular, and radial nerve origin were normal in all 37 cadavers.

## 4. Discussion

Posterior cord of brachial plexus, usually formed by union of posterior divisions of upper, middle, and lower trunk, respectively. In their study, Fazan et al. [4] observed that the posterior cord was formed by posterior division of upper and middle trunk (there was no lower trunk) in 9% of brachial plexus however Chaudhary et al. [5] reported the presence of four trunks in brachial plexus, namely, I, II, III, and IV in 3 cases of upper extremities and in all the three cases posterior cord was formed by union of posterior division of I, II, and III trunk having root value C_5_–C_8_.There were also reports of cases where ventral rami of C_5_ and C_6_ did not join to form upper trunk dividing independently into anterior and posterior divisions [6, 7]. In the present study we also observed C_5_ and C_6_ ventral rami dividing independently into anterior and posterior divisions (there was no upper trunk) the posterior divisions of C_5_ and C_6_ then united with the posterior divisions of middle and lower trunk to form posterior cord in 16.2% of upper extremities. Such variations in which there was no upper trunk may increase the chance of nerve root avulsion due to downward traction injury of brachial plexus [8]. 

These variations can be correlated embryologically. Position and width of a limb bud determine its innervations; limb bud is supplied by nerves of region where it is implanted. Segregation of the developing structure within the limb direct growing nerve fibers (axons) and determines their grouping into bundles leading to formation of roots and trunks [9]. As the expression of chemoattractants and chemorepulsants regulates the growing nerve fibers (axons) in a highly coordinated site-specific fashion, any alterations in signaling between the mesenchymal cells and neuronal growth cones can lead to significant variations [10]. In the present study, the nonformation of the upper trunk appears to be a result of overexpression of chemoattractants/repulsants, leading to separation of the C_5_ and the C_6_ roots. Miller in different vertebrates observed patterns of the roots, trunks, cords, and branches of brachial plexus and reported no trunk formation in amphibians, reptiles, and dogs [11] in our study also in 16.2% cases upper trunk was not formed, which partially fits into this category and indicates ontogeny repeats phylogeny.

 According to standard text books of anatomy the posterior cord is related laterally to the second part of axillary artery [1]. There were reports where posterior cord was present lateral to the second part of axillary artery [12, 13]. In our study we also observed it was lateral to the second part of axillary artery in 18.9% cases. 

The branches of posterior cord of brachial plexus include upper subscapular, thoracodorsal, lower subscapular, axillary, and radial nerve. Bhat and Girijavallaban et al. [3] and Jamuna [14] described case where posterior cord split into anterior and posterior divisions. The axillary nerve in both cases took origin from posterior division of posterior cord. However Matejcik [15] and Chaudhary et al. [5] in their study on brachial plexus observed axillary nerve originating from posterior division of upper trunk in three cases and one case, respectively. Axillary nerve was taking origin from posterior division of upper trunk in 10.8% upper extremities in the present study which is much higher than Matejcik and Chaudhary et al. On the other hand we also observed thoracodorsal nerve originates from axillary nerve in 22.9% upper extremities which was much higher than previous studies of Fazan et al. (13%) [4] and Muthoka et al. (10.3%) [16].

We believe that prior knowledge of the previously mentioned variations of posterior cord of brachial plexus is useful to surgeons for surgical treatment of tumor of nerve sheath such as schwannomas and neurofibromas, anesthesiologists for administering local anesthetic blocks, and clinicians for interpreting effects of nerve injuries to the upper limb.
